# Characterization of human placental fetal vessels in gestational diabetes mellitus

**DOI:** 10.1007/s00424-024-03028-6

**Published:** 2024-10-09

**Authors:** Philine S. Carstens, Heike Brendel, M. Leyre Villar-Ballesteros, Jennifer Mittag, Clara Hengst, Coy Brunssen, Cahit Birdir, Paul D. Taylor, Lucilla Poston, Henning Morawietz

**Affiliations:** 1 Division of Vascular Endothelium and Microcirculation, Department of Medicine III, Faculty of Medicine and University Hospital Carl Gustav Carus, TUD Dresden University of Technology, Fetscherstr. 74, 01307 Dresden, Germany; 2 Department of Obstetrics and Gynecology, Faculty of Medicine and University Hospital Carl Gustav Carus, TUD Dresden University of Technology, Dresden, Germany; 3 Center for Feto/Neonatal Health, Faculty of Medicine and University Hospital Carl Gustav Carus, TUD Dresden University of Technology, Dresden, Germany; 4 Department of Women & Children’s Health, School of Life Course & Population Sciences, King’s College London, London, UK

**Keywords:** Gestational diabetes, Fetal placental vessels, Endothelial function, Substance P

## Abstract

Gestational diabetes mellitus is one of the most common complications during pregnancy. Its prevalence is rapidly increasing worldwide. Gestational diabetes mellitus is leading to an elevated risk for the development of endothelial dysfunction and cardiovascular diseases both in the mother and the child in later life. The underlying pathophysiological mechanisms are not well-understood. Therefore, we aimed to characterize the endothelial function in fetal placental vessels from mothers with gestational diabetes mellitus. In this study, we distinguished between insulin-treated and diet-controlled gestational diabetes mothers and compared them to a normoglycemic control group. The clinical data confirmed pre-conceptional overweight as a risk factor in women with insulin-treated gestational diabetes mellitus. The insulin-treated gestational diabetes group was also characterized by a recent family history of diabetes compared to mothers of the control or diet-controlled gestational diabetes group. Analyses of blood serum from umbilical cords suggested a reduced fetal insulin metabolism in the insulin-treated gestational diabetes group. Vascular function analysis in fetal placental vessels revealed an altered substance P-induced vasorelaxation in vessels from patients with insulin-dependent gestational diabetes. Inhibition of nitric oxide synthase affected only fetal vessel segments from the control group or diet-controlled gestational diabetes group, but not from insulin-dependent gestational diabetes. Finally, we found a significantly decreased substance P receptor (*TACR1*) mRNA expression in fetal vessel segments from patients with insulin-treated gestational diabetes. In conclusion, we provide evidence that different pathophysiological mechanisms might be responsible for the development of insulin-treated versus diet-controlled gestational diabetes. Only in fetal vessels from patients with insulin-treated gestational diabetes were we able to detect an endothelial dysfunction and a reduced fetal insulin conversion. This provides novel insights into the pathophysiology of the subtypes of gestational diabetes.

**Supplementary Information:**

The online version contains supplementary material available at 10.1007/s00424-024-03028-6.

## Introduction

The prevalence of gestational diabetes mellitus (GDM) is alarmingly increasing worldwide [1]. In a recent meta-analysis, the global standardized prevalence of GDM was 14.0% with a regional prevalence between 7 and 27% [2]. The pathophysiology of gestational diabetes is not well-understood [3]. The global obesity epidemic, a family history of diabetes, an increased maternal age, and an inactive lifestyle are discussed as possible risk factors [4, 5]. It is suspected that GDM is caused by ß-cell dysfunction in the background of an existing insulin resistance [6]. These pathophysiological ß-cell states are likely triggered by a persistent abundance of energy, leading to excessive insulin production and secretion by the pancreatic ß-cells. They are physiologically not designed to carry this constantly increased burden, resulting in functional overload and finally ß-cell dysfunction [7]. In a gestational diabetic pregnancy, women experience a 50–60% decrease in insulin sensitivity with advancing gestation [8]. This decreased insulin sensitivity during pregnancy affects the glucose transfer to the growing fetus [3]. Thus, pregnancy is considered a vulnerable time for metabolic dysfunction. The ß-cells of a healthy pregnant woman increase insulin production through hypertrophy and hyperplasia to maintain normoglycemia. This is not possible in a gestational diabetic pregnancy due to ß-cell dysfunction. The increased circulating blood glucose concentration cannot be compensated. A vicious cycle of hyperglycemia, increased insulin resistance, and pancreatic ß-cell dysfunction develops [3]. The interplay between hyperglycemia and failure of pancreatic ß-cells can lead to glucotoxicity and vascular dysfunction [9]. Furthermore, GDM can induce inflammation and oxidative stress [10]. Oxidative stress with an increased formation of reactive oxygen species (ROS) can cause endothelial dysfunction and atherosclerosis [11, 12]. Endothelial dysfunction further exacerbates the deleterious effects of diabetes-induced hyperglycemia [13].

Intra-uterine programming of disease in GDM may result in short- and long-term metabolic alterations and increased cardiometabolic risk of mother and child throughout life [14]. GDM can cause fetal hyperinsulinemia, which might lead to endothelial dysfunction in the fetal macro- and microcirculation. Hyperinsulinemia is also associated with accelerated weight gain up to 2 years and increased adipogenic activity and risk of obesity [15]. Another consequence is the development of diabetic fetopathy, increasing the fetal risk for macrosomia, premature birth, and respiratory distress syndrome [16]. Women with a history of GDM have also an increased risk to develop hypertension [17]. Even when maternal glucose tolerance normalizes postpartum, the risk for fetal and maternal long-term consequences like the manifestation of the metabolic syndrome persists. The metabolic syndrome is a global health problem with rapidly increasing prevalence, characterized by an accumulation of various health issues including obesity, hypertension, dyslipidemia, pathological glucose tolerance, endothelial dysfunction, and the development of cardiovascular diseases [5].

The treatment options for GDM are very limited. In some patients, insulin therapy is prescribed to restore normoglycemia. However, the effectiveness of this therapy is controversial [18]. The only intervention that might mediate possible health improvements for women and their babies are lifestyle changes (including as a minimum healthy eating, physical activity, and self‐monitoring of blood sugar levels) [18]. Based on these therapeutic options, women with GDM have usually insulin-treated gestational diabetes (iGDM) or diet-controlled gestational diabetes (dGDM). The detailed pathophysiological mechanism of these two subtypes of GDM is largely unknown. Furthermore, the impact of iGDM or dGDM on the endothelial function in human placental fetal vessels is not well-understood.

Therefore, we expected differences in the clinical data and in the analysis of umbilical cord blood between mothers with iGDM, dGDM, or a normoglycemic control group. In addition, we hypothesized that the two GDM subtypes might differentially affect fetal placental vessel function.

## Materials and methods

### Clinical data

Patients were admitted to the Department of Obstetrics and Gynecology, University Hospital Carl Gustav Carus, TUD Dresden University of Technology, Dresden, Germany. All patients received detailed verbal and written information about the study and gave written informed consent. The study was approved by the Ethics Committee of the TUD Dresden University of Technology (EK 277–07-2018 (BO), extension 2022). The investigation conforms with the principles outlined in the Declaration of Helsinki. Clinical characteristics of patients are listed in the “Results” section in Table 1. Due to their clinical characteristics, the participants were assigned to one of the following groups: normoglycemic control group (CG), insulin-treated gestational diabetes (iGDM), or diet-controlled gestational diabetes (dGDM).

**Table 1:** Clinical characteristics of study participants of the normoglycemic control group (CG), the insulin-treated (iGDM), and the diet-controlled gestational diabetes (dGDM) group

Characteristics	CG	iGDM	dGDM	*p*
Total *n* = 77	55	12	10	
General anamnesis				
Age (years)	33.09 ± 5.47	34.17 ± 5.39	35.40 ± 4.45	0.4185
Week of pregnancy	38.41 ± 1.47	38.56 ± 0.86	38.91 ± 0.80	0.5380
BMI (before pregnancy) (kg/m^2^)	26.85 ± 5.50	35.85 ± 9.18	24.05 ± 3.30	0.00003*
Abortion/EUP (*n*)	0.51 ± 0.79	0.33 ± 0.65	0.60 ± 1.35	0.7466
IVF/ICSI (yes:no; % total)	4:50; 7.41%	2:10; 16.67%	0:10; 0%	0.3471
Birth mode (caesarean section yes:no; % total)	51:3; 94.40%	12:0; 100%	10:0; 100%	0.5337
Nicotine abuse (yes:no; % total)	3:51; 5.56%	1:11; 8.33%	0:10; 0%	0.6766
Drug abuse (yes:no; % total)	2:52; 3.7%	0:12; 0%	0:10; 0%	0.6617
Clinical data of the mothers				
Essential hypertension (yes:no; % total)	9:45; 16.67%	3:9; 25%	0:10; 0%	0.2676
Pregnancy-induced hypertension (yes:no; % total)	1:53; 1.85%	1:11; 8.33%	0:10; 0%	0.3876
COVID-19 infection (yes:no; % total)	9:45; 16.67%	0:12; 0%	0:10; 0%	0.1284
Mental disorders (yes:no; % total)	4:50; 7.41%	3:9; 25%	1:9; 10%	0.2032
Thyroid disorders (yes:no; % total)	8:46; 14.81%	4:8; 33.33%	1:9; 10%	0.2531
Asthma (yes:no; % total)	6:48; 11.11%	1:11; 8.33%	0:10; 0%	0.5373
Neurodermatitis/psoriasis (yes:no; % total)	6:48; 11.11%	1:11; 8.33%	0:10; 0%	0.5373
Medication				
Aspirin (yes:no; % total)	12:42; 22.22%	4:8; 33.33%	1:9; 10%	0.4296
Anti-hypertensive therapy (yes:no; % total)	2:52; 3.70%	1:11; 8.33%	0:10; 0%	0.6021
Iron supplementation (yes:no; % total)	10:44; 18.52%	1:11; 8.33%	0:10; 0%	0.2547
l-Thyroxine (yes:no; % total)	7:47; 12.96%	4:8; 33.33%	1:9; 10%	0.1911
Aerosol therapy for asthma (yes:no; % total)	5:49; 9.26%	1:11; 8.33%	0:10; 0%	0.6110
Family history				
Diabetes mellitus (yes:no; % total)	16:39; 29.09%	7:5; 58.33%	0:10; 0%	0.0123*
Hypertension (yes:no; % total)	18:37; 32.73%	2:10; 16.67%	0:10; 0%	0.0712
Fetal characteristics				
Fetal sex (female:male)	(30:24)	(7:5)	(3:7)	
Birth weight (g)	3197.54 ± 539.99	3491.25 ± 529.45	3337.00 ± 366.70	0.1927
Umbilical cord pH	7.31 ± 0.05	7.31 ± 0.06	7.31 ± 0.02	0.9023
Apgar score 1 min	9.00 ± 0.59	8.83 ± 0.94	9.10 ± 0.32	0.5913
Apgar score 5 min	9.58 ± 0.60	9.33 ± 0.78	9.80 ± 0.42	0.2074
Apgar score 10 min	9.85 ± 0.36	9.67 ± 0.65	10.00 ± 0.00	0.1496
IUGR/SGA (yes:no; % total)	4:50; 7.41%	1:11; 8.33%	0:10; 0%	0.6657
Macrosomia (yes:no; % total)	2:52; 3.70%	2:10; 16.67%	0:10; 0%	0.1425

*BMI* body mass index, *CG* control group, *dGDM* diet-controlled gestational diabetes group, *EUP* extrauterine pregnancy, *FE* iron (ferric) supplementation, *ICSI* intracytoplasmic sperm injection, *iGDM* insulin-treated gestational diabetes group, *IUGR* intrauterine growth restriction, *IVF* in vitro fertilization, *SGA* small for gestational age

### Fetal vessel sampling

From each placenta, we isolated up to six fetal vessels. The maternal side of the placenta was turned upwards, the decidual tissue was carefully removed, and the vascular tree of the chorionic villus was examined. The corresponding vessels were excised, carefully separated from surrounding tissue, rinsed with phosphate-buffered saline (PBS), and thoroughly checked under the microscope for proper preparation. For vascular function analysis, up to four vessel segments were analyzed in parallel with different substances. For RNA isolation, two vessel rings of each placenta were rinsed with ice-cold PBS, pooled, and snap-frozen in liquid nitrogen for further analysis.

### Functional measurements

The functional measurements were performed using a DMT Multi Wire Myograph System—Model 620 M (Danish Myo Technology A/S, Hinnerup, Denmark). Several fetal vessels from each placenta were carefully isolated. Isolated fetal vessel segments were washed in physiological salt solution (PSS) before mounting on pins for isometric tension recordings. The organ bath contained a physiological salt solution bubbled with 5% CO_2_ in O_2_. After normalization and equilibration, vasoconstriction of fetal blood vessels was induced twice by 123 mM potassium chloride. After several washing steps, serotonin-induced contraction was tested (1 nM to 30 µM). To study vasorelaxation, fetal vessel rings were precontracted with 10 µM serotonin and relaxed by increasing concentrations of substance P (1 to 30 nM). In addition, fetal vessel rings were precontracted with serotonin, and relaxation was analyzed by increasing concentrations of insulin (1 to 30 nM). After the final dose of substance P or insulin, a 25-min incubation time was recorded to observe stable vasorelaxing effects. Subsequently, 300 mM L-NAME was added to each organ chamber of the Mulvany myograph and incubated for 30 min. Afterwards, concentration–response curves for substance P and insulin were repeated. Sodium nitroprusside (SNP) (1 nM to 100 µM) was added to precontracted vessels to analyze the endothelium-independent relaxation. Finally, we tested noradrenalin and U46619 for their contractile properties in fetal vessels. Acetylcholine and bradykinin were tested in precontracted fetal vessel segments for their vasorelaxing properties.

### Real-time PCR

The vessel segments were isolated from the human placenta and shock-frozen in liquid nitrogen for further analyses. Tissue samples were homogenized with a Precellys homogenizer (VWR, Peqlab, Erlangen, Germany). Total RNA was isolated using peqGOLD (VWR, Peqlab, Erlangen, Germany). Reverse transcription of mRNA into cDNA was performed with SuperScript II Reverse Transcriptase according to manufacturer’s instructions (Thermo Fisher Scientific, Waltham, MA, USA) using 500 ng total RNA and random hexamer primers. Quantification was performed by real-time PCR with GoTaq qPCR Master Mix (Promega, Mannheim, Germany). Analysis of raw data was performed with 7500 Software Version 2.06 (Applied Biosystems by Life Technologies, Darmstadt, Germany). Evaluation of the data was done using a mathematical model of relative expression ratio in real-time PCR under constant reference gene expression [19]. The primers are listed in Table 2.

**Table 2:** List of primers. The primers (Sigma-Aldrich) were used at an annealing temperature of 60 °C

Name	Species	Sequence (5′-3′)
*ADRA1A* sense	Human	GGAGAAGAAAGCGGCCAAAA
*ADRA1A* antisense	Human	TCAGAGGGCTTGAAATCAGGG
*BDKRB1* sense	Human	CCAACTACAGTTGTGAACGCC
*BDKRB1* antisense	Human	AGGATGATGCCATGCACAGT
*CHRM3* sense	Human	AGCTAAGGTACAATAAGGTTTTGCT
*CHRM3* antisense	Human	ACAGAGGAGTGGACCAGATGT
*eNOS* sense	Human	GAACCTGTGTGACCCTCACCCC
*eNOS* antisense	Human	TGGCTAGCTGGTAACTGTGC
*HTR2B* sense	Human	CAACGAAACCAGAGGGGGAA
*HTR2B* antisense	Human	AAGCTCAGTGAAAGAAACTGATAGC
*INSR* sense	Human	ACCGCTTTACGCTTCTTCAA
*INSR* antisense	Human	TGAGGAACTCAATCCGCTCT
*TACR1* sense	Human	AGCAGAGTCGTGTGCATGAT
*TACR1* antisense	Human	GTCACACAGATGTGGTACACTTTC
*TBP* sense	Human	CGCCGGCTGTTTAACCTTCG
*TBP* antisense	Human	AGAGCATCTCCAGCACACTC

*ADRA1A* alpha1 A-adrenergic receptor, *BDKRB1* bradykinin receptor B1, *CHRM3* M3 muscarinic acetylcholine receptor, *eNOS* endothelial NO synthase, *HTR2B* 5-HT(2B) serotonin receptor, *INSR* insulin receptor, *TACR1* tachykinin-1 receptor, *TBP* TATA-box binding protein

### Histology of placental sections

Tissue samples were fixed in PBS-buffered paraformaldehyde (4% PFA) in embedding cassettes to preserve the tissue architecture as naturally as possible. Prior to further processing, the sections were placed in an embedding machine overnight. This dehydrated the tissue through an ascending ethanol series. The tissue was then incubated in an intermediate medium (xylene). Subsequently, the now fluid-free tissue samples were infiltrated with liquid paraffin and poured into paraffin blocks. Three micrometers serial sections of the placental tissue was made on a rotation microtome (Leica Microsystems, Wetzlar, Germany) and mounted on glass slides. The routine staining with hematoxylin and eosin, staining cell nuclei blue, erythrocytes red, and cytoplasm pink was performed. All stained sections were acquired using a Zeiss Axio Scan.Z1 slide scanner (Carl Zeiss Microscopy GmbH, Jena, Germany).

### Serum blood analysis

The fetal blood samples were obtained from umbilical vein and arteries. After centrifugation, the blood serum was isolated. Serum glucose, insulin, and C-peptide concentrations were measured in the Institute of Clinical Chemistry and Laboratory Medicine, University Hospital Carl Gustav Carus, TUD Dresden University of Technology, Dresden, Germany.

### Data handling and statistical analysis

Data are shown as mean ± standard deviation (SD). Normal (Gaussian) distribution was tested by the Shapiro–Wilk normality test. As a non-parametric test, Kruskal–Wallis and Dunn’s multiple comparison tests were performed. Gaussian distributed data were analyzed with a *t*-test or one-way ANOVA followed by Bonferroni post hoc test, respectively (GraphPad Prism 8–10, GraphPad Software, Inc., La Jolla, USA). A value of *p* < 0.05 was considered statistically significant.

## Results

### Clinical data

The clinical characteristics of study participants of the normoglycemic control group (CG), insulin-treated gestational diabetes (iGDM) group, and diet-controlled gestational diabetes (dGDM) group are presented in Table 1.

**Fig. 1:**
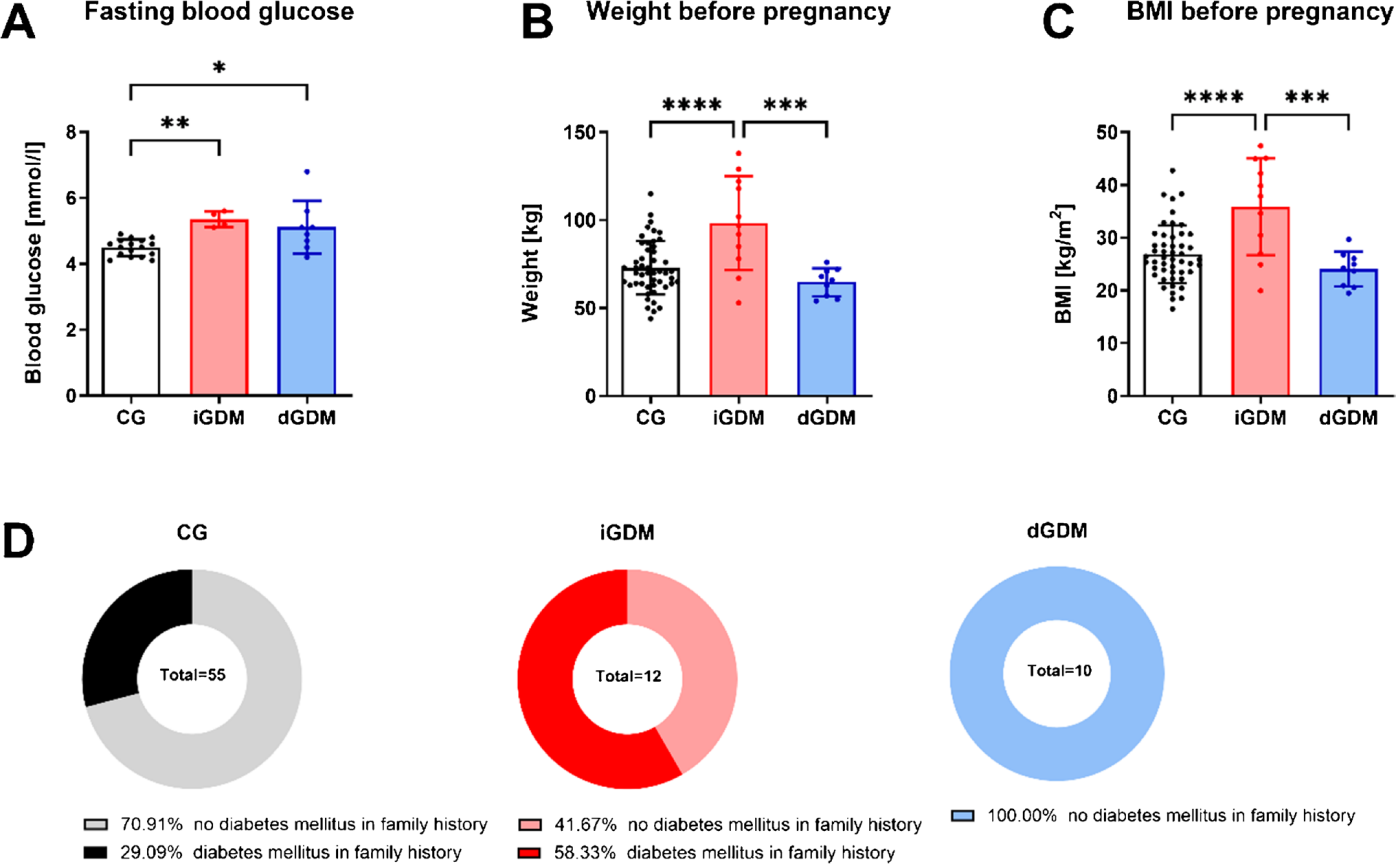
Differences in clinical data and family anamnesis of insulin-dependent gestational diabetes mellitus (iGDM). **A** Fasting blood glucose levels in pregnancy of study participants. Statistics: one-way ANOVA; *p* < 0.05; *n* = 8–17. **B** Weight and (**C**) BMI before pregnancy of women in the control group (CG), patients diagnosed with insulin-treated gestational diabetes mellitus (iGDM), and diet-controlled GDM (dGDM). Statistics: one-way ANOVA; *p* < 0.05; *n* = 9–49. **D** Percentages of reported cases with a family history of diabetes mellitus. The control group was compared with the insulin-dependent (iGDM) as well as the diet-controlled women with gestational diabetes (dGDM)

Significant differences were observed in three criteria between the groups (Fig. 1). First, the fasting blood glucose levels of pregnant women were significantly elevated in both groups of gestational diabetic patients compared to normoglycemic control (Fig. 1A). Because the 75 g OGTT is the standard diagnostic test, this ensured the involvement of validated GDM patients in our study.

Furthermore, the weight and BMI of the patients and their family history of diabetes showed significant differences between the groups. The weight of women before pregnancy was significantly higher in the insulin-treated gestational diabetic group compared to the control group and the diet-controlled group of gestational diabetics (Fig. 1B). This is supported by the BMI before pregnancy (Fig. 1C). While the iGDM women have the highest BMI before pregnancy, the dGDM women tend to have the lowest.

When analyzing the family history of parents, grandparents, and siblings of the patients, significantly more diabetic cases were found in the iGDM group. Fifty-eight percent of the studied iGDM women had a family history of diabetes mellitus. In contrast, none of the diet-controlled gestational diabetes patients reported other cases of diabetes mellitus in their families (Fig. 1D).

### Serum analysis of umbilical cord blood

**Fig. 2:**
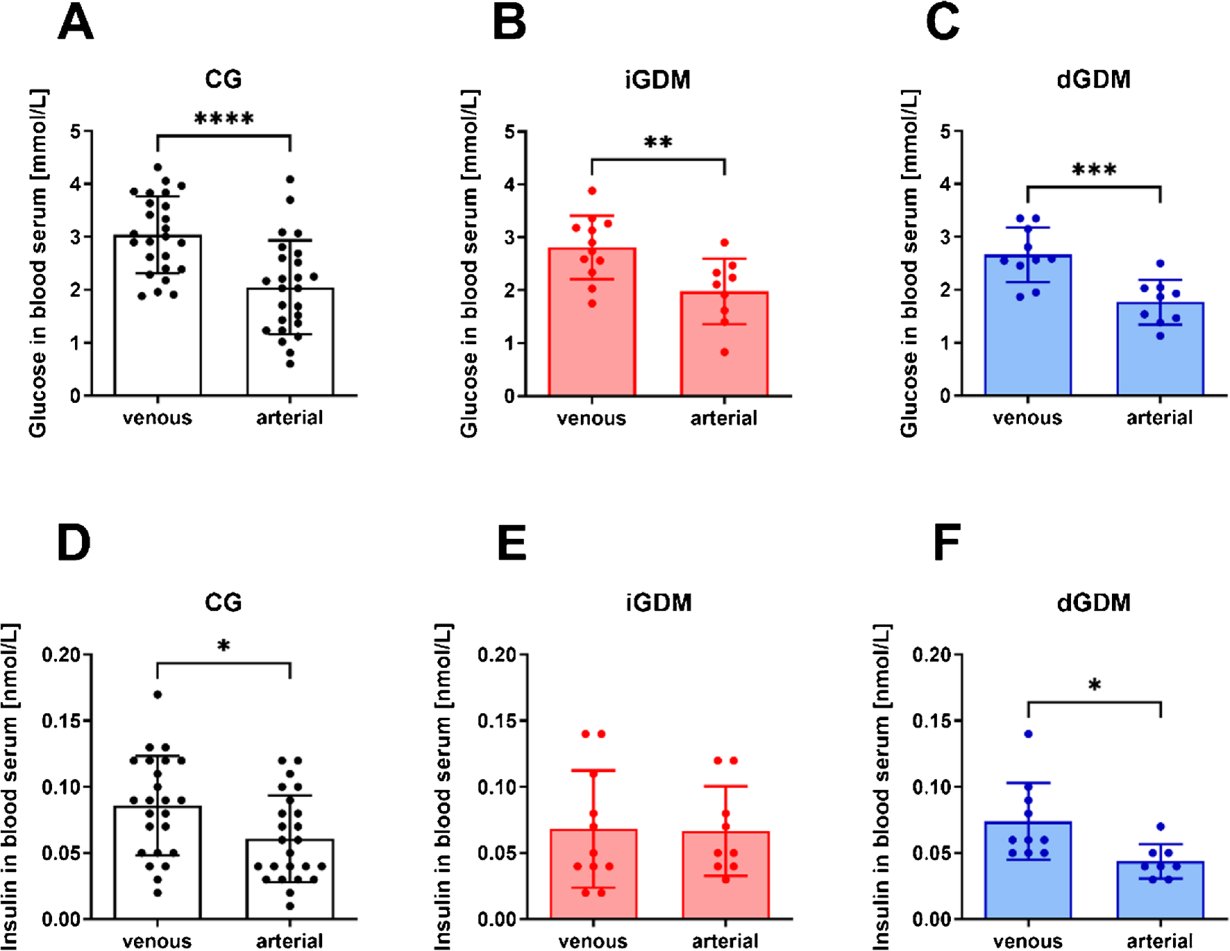
Glucose levels and insulin concentrations in venous and arterial fetal blood. Arterial and venous level of glucose in (**A**) serum of umbilical cord blood in control group (CG), **B** serum of umbilical cord blood in insulin-treated (iGDM), and (**C**) diet-controlled (dGDM) gestational diabetes mellitus patients. Statistics: *t*-test; *p* < 0.05; n = 8–25. Arterial and venous level of insulin in (**D**) serum of umbilical cord blood in control group (CG), **E** serum of umbilical cord blood in insulin-treated (iGDM), and (**F**) diet-controlled (dGDM) gestational diabetes mellitus patients. Statistics: *t*-test; *p* < 0.05; *n* = 8–25

The blood glucose level of the venous blood coming from the mother to supply oxygen and nutrients to the infant and the arterial carrying waste products from the fetus did not differ between the study groups. Interestingly, the arterial umbilical cord blood contained significantly less glucose than the venous in all three study groups (Fig. 2A–C). Several monosaccharides had higher concentrations in the arterial cord plasma, while amino acids were higher in venous plasma, suggesting that the main differences in the measured arterial and venous plasma metabolomes are related to amino acid and energy metabolism [20]. This allowed the first insights into the fetal glucose metabolism.

In contrast to glucose circulating in the cardiovascular system, insulin is not able to pass the placental barrier [21]. Thus, an exchange of insulin between the mother and her unborn child is not possible. In this study, the examined blood was taken from the umbilical vein and arteries. The obtained blood samples consisted of venous blood that had passed the intervillous space and arterial blood that had not yet passed it. Accordingly, the following results shown in Fig. 2D, E represent the insulin concentrations synthetized from the ß-cells of the fetal pancreas. Thus, a conclusion can be drawn about the fetal insulin metabolism. The examined fetal blood of the normoglycemic control as well as the diet-controlled gestational diabetes patients showed significantly lower arterial insulin levels compared to the venous (maternal) serum (Fig. 2D, F). However, in the fetal blood of mothers with insulin-treated gestational diabetes, no significant difference between venous and arterial insulin concentrations was detectable, indicating an insulin resistance (Fig. 2E). Concentrations of C-peptide that is able to cross the placenta showed no significant differences between the study groups or venous and arterial blood (Online Resource, Fig. 1A–C).

### Functional analysis revealed alterations in fetal vessels of insulin-dependent gestational diabetic mothers

**Fig. 3:**
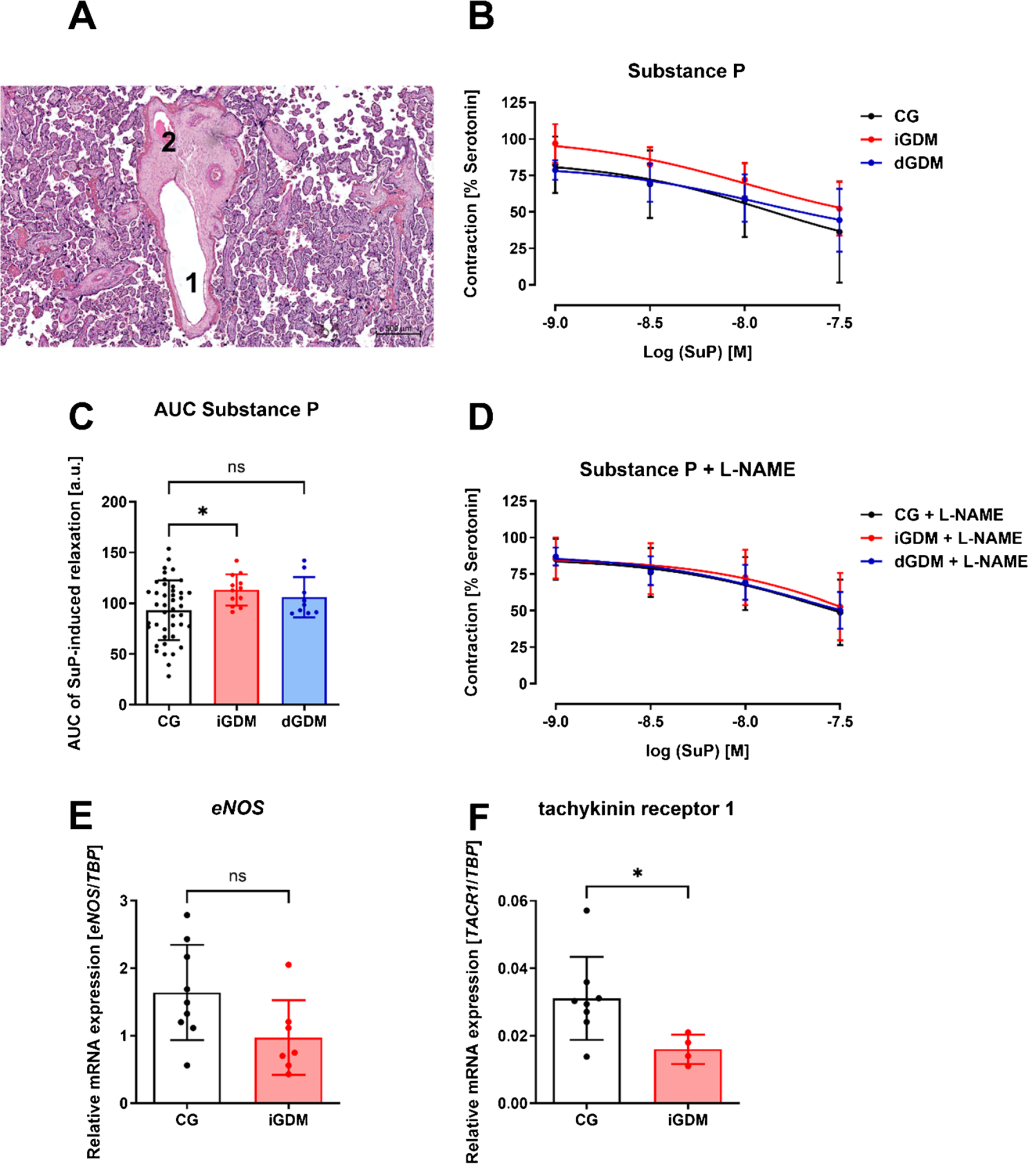
Substance P–induced vasorelaxation in fetal placental vessels. **A** Representative histological image of a mature fetal villus with its blood vessels: 1 indicates a fetal vein and 2 indicates a fetal artery. **B** Dose–response curves of cumulative application of substance P in precontracted fetal placental vessels from control (CG) mothers with insulin-treated gestational diabetes (iGDM) and diet-controlled gestational diabetes (dGDM); *n* = 8–43. **C** Area under the curve of dose–response curves of substance P in fetal vessels from CG, iGDM, and dGDM. Statistics: one-way ANOVA; *p* < 0.05; *n* = 8–43. **D** Dose–response curves of substance P after application of L-NAME in fetal vessels from CG, iGDM, and dGDM; *n* = 8–40. **E** Relative *eNOS* mRNA expression in fetal placental vessels from the normoglycemic control group (CG) and insulin-treated gestational diabetes group. Statistics: unpaired *t*-test; *p* < 0.05; *n* = 7–10. **F** Relative *TACR1* mRNA expression in fetal placental vessels from the normoglycemic control group (CG) and insulin-treated gestational diabetes group. Statistics: unpaired *t*-test; *p* < 0.05; *n* = 4–8

A representative histological image of the mature fetal villus with its blood vessels analyzed in this study is shown in Fig. 3A. In serotonin-precontracted fetal vessels, we were able to induce vasorelaxation with substance P (Fig. 3B). The analysis of the area under the curve revealed a significantly increased area in patients with insulin-treated gestational diabetes compared to the control group (Fig. 3C). Application of nitric oxide inhibitor L-NAME to the vascular segments abolished the differences in substance P–induced vasorelaxation between the three study groups (Fig. 3D). By stimulation of the endothelial NK1 receptor (encoded by the tachykinin receptor 1; *TACR1*), substance P is known to induce a nitric oxide-mediated endothelium-dependent vasorelaxation [22]. The observation that an inhibition by L-NAME was only detectable in the control group and the diabetic-treated gestational diabetes group might indicate a lower nitric oxide-mediated component of endothelium-dependent relaxation in the vessels of insulin-treated gestational diabetic mothers. Nitric oxide synthase *eNOS* mRNA expression tended to be reduced in fetal vessels of the insulin-treated gestational diabetic group without reaching significance (Fig. 3E). The expression of *TACR1* was significantly reduced in the iGDM group (Fig. 3F).

**Fig. 4:**
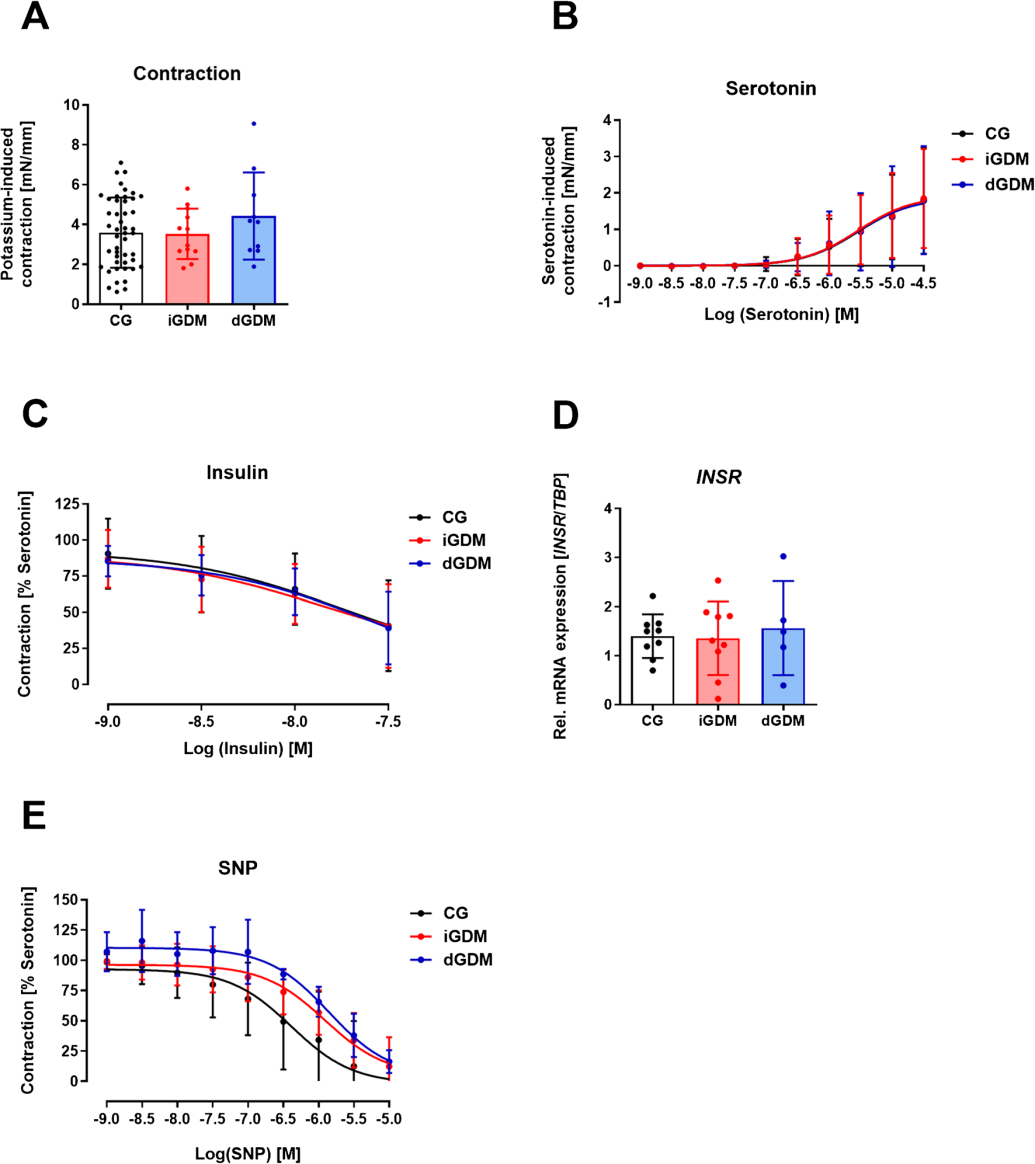
Vascular analysis of fetal placental vessels. **A** Potassium chloride (KCl)–induced contraction in fetal vessels of normoglycemic controls (CG), insulin-treated gestational diabetic mothers (iGDM), and diet-controlled gestational diabetic mothers (dGDM); *n* = 10–47. **B** Concentration–response curves of serotonin-induced vasoconstriction in fetal placental vessels of CG, iGDM, and dGDM; *n* ≥ 10. **C** Concentration–response curves of insulin in fetal placental vessels of CG, iGDM, and dGDM; *n* ≥ 10. **D** Relative mRNA expression of insulin receptor *INSR* in fetal vessels of CG, iGDM, and dGDM; *n* = 5–10. **E** Concentration–response curve of sodium nitroprusside (SNP) in fetal vessels from CG, iGDM, and dGDM; *n* ≥ 3

In addition to substance P, several others potentially vasoactive substances were tested in the characterization of vascular function in fetal placental vessels. The potential of potassium chloride, serotonin, norepinephrine, and U46619 to induce vasoconstriction in fetal vessels was tested. High concentrations of potassium chloride induced an endothelium-independent vasoconstriction of blood vessels (Fig. 4A). This happens through the opening of voltage-dependent Ca^2+^ channels in the vessel wall leading to a depolarization of the vascular smooth muscle. This results in the contraction of the smooth muscle cells in the vessel wall. In Fig. 4A, the maximum effect of a single dose of 123 mM KCl in the fetal vessels of the placenta is shown. A significant contraction was notable which did not differ between normoglycemic and hyperglycemic fetal vessels. The tissue hormone and neurotransmitter serotonin were tested in dose–response curves (Fig. 4B). Serotonin induced vasoconstriction in fetal vessels but showed no differences between the three study groups. The neurotransmitter norepinephrine is a well-described vasoconstrictor in several other human vessels. However, norepinephrine was not able to induce a vasoconstriction in fetal placental vessels (Online Resource, Fig. 2A). In contrast, the thromboxane analog U46619 induced a vasoconstriction in fetal vessels of the placenta (Online Resource, Fig. 2B). However, it was not possible to reverse the contractile effects on the vessel segments by washing. Therefore, U46619 was not used for further functional analysis.

Vasorelaxation was tested in serotonin-precontracted vessel segments. Besides substance P, we tested insulin, sodium nitroprusside, acetylcholine, and bradykinin. Application of insulin to fetal vessel segments induced a vasorelaxation (Fig. 4C). However, no significant differences in insulin-induced vasorelaxation were detected between the study groups. Furthermore, the mRNA expression of the insulin receptor *INSR* was not different between the study groups (Fig. 4D). The nitric oxide donor sodium nitroprusside was also able to induce vasorelaxation in fetal vessels (Fig. 4E). This endothelium-independent vasorelaxation was not significantly different between the groups.

In addition, vasorelaxation with acetylcholine and bradykinin was tested. Acetylcholine was not able to induce a relaxation in the fetal vessels (Online Resource, Fig. 2C). Similarly, bradykinin has a potent endothelium-dependent vasodilatory effect in several other human vessels. However, in the fetal vessels of the placenta, bradykinin did not cause a relaxation (Online Resource, Fig. 2D).

## Discussion

In this study, we first evaluated the impact of different risk factors on gestational diabetes mellitus (GDM). The fasting blood glucose level in the oral glucose tolerance test (OGTT) is an independent predictor of GDM [23]. Our results confirm that both GDM groups have significantly higher fasting blood glucose levels than the control group in the OGTT.

The pathomechanisms of diet-controlled and insulin-treated gestational diabetes are not well-understood [24]. We provide evidence for a potential link between insulin-controlled GDM and obesity. Obesity is a main risk factor of diabetes mellitus, and its prevalence is rising in the recent decades [25]. This is highlighted by the term “Diabesity” combining the deleterious effects of diabetes and obesity on human health [26]. Interestingly, we did not observe a correlation between the pre-conceptional body weight and the development of diet-controlled gestational diabetes (dGDM). A possible explanation might be an overall healthier lifestyle of women with a pre-conceptually lower body weight also reducing the risk for the development of GDM [27]. Therefore, the origin of dGDM might involve additional risk factors other than obesity. In contrast, iGDM is likely to develop in the context of adipose tissue–associated insulin resistance [23].

A genetic predisposition to diabetes may also underlie an exaggerated response of the maternal metabolism to excess adiposity, exceeding the threshold for the development of GDM [8]. In our cohort, only the patients with insulin-treated gestational diabetes showed indications for a genetic predisposition, as there were more cases with a family history of diabetes mellitus. In contrast, no family member of the women with dGDM had diabetes mellitus. This further supports the concept of different pathomechanisms underlying the two GDM subtypes.

In iGDM patients, an adipose tissue–associated insulin resistance seems to be responsible for the reduced insulin sensitivity in peripheral target tissues. In women with diet-controlled gestational diabetes, a centrally decreased insulin secretion already appears in the postprandial phase, indicating a dysfunction of ß-cells in this group. Both pathomechanisms exacerbate hyperglycemia and can act synergistically [28] or independently [29].

The blood samples of this study were obtained by puncturing the vessels of the umbilical cord. Therefore, the venous blood has already perfused the intervillous space and crossed the placental barrier, and the exchange of oxygen and nutrients has already taken place. Since the fetus lacks the necessary enzymes for its own gluconeogenesis, glucose enters the fetal circulation exclusively via maternal blood [30]. The arterial blood samples represent the status before the passage of the placental barrier. The removal of CO_2_ and waste products from the fetal circulation has not yet occurred. Even while some studies found evidence for altered glucose levels in the umbilical cord blood of patients with gestational diabetes [31], the data from our patients could not confirm these findings. There was no significant difference in the glucose levels between control and GDM groups. This could be due to an optimized maternal GDM therapy or a limited transfer of glucose via the placental barrier to the fetus in a hyperglycemic maternal metabolic situation [32].

The insulin measured in the umbilical cord blood in our study is solely synthesized by the fetus since insulin cannot pass the placenta barrier. There were no signs of fetal endogenous hyperinsulinemia or increased fetal birth weight due to maternal GDM in our study. Both findings suggest that fetal ß-cells of hyperglycemic patients remained intact in this study. This might be due to an optimized therapy of women with gestational diabetes. Adequate therapy can significantly reduce the risk of fetal macrosomia with its clinical consequences [33]. Furthermore, this study suggests that less insulin was metabolized in fetuses of the iGDM group compared to the other study groups. This is another indication that the disease profiles of the two GDM subtypes might differ. Pivovarova et al. also found a close relationship between impaired insulin breakdown in the fetal liver and the development of a metabolic syndrome. They suggest a reduced insulin clearance in the fetal liver before further disturbances in glucose metabolism develop [34]. A decreased insulin metabolism leads to chronically elevated levels of circulating insulin that can further promote the development of insulin resistance [35].

Especially interesting is the metabolic difference in fetuses and mothers with insulin-dependent GDM. In extreme diabetes complications (such as diabetic ketoacidosis), there are important metabolic changes partly mediated by the renal handling of amino acids [36]. Extreme pathologic conditions and corresponding models may help to understand the metabolic changes in GDM [37].

The number of studies analyzing the concentrations of C-peptide during pregnancy is still limited. Furthermore, the results of these studies are partially controversial. However, C-peptide might be an excellent biomarker of insulin secretion and ß-cell function [38]. Higher levels of C-peptide are associated with improved ß-cell function. Furthermore, C-peptide has own physiological effects on endothelial function and might participate in the inhibition of micro- and macrovascular complications of diabetes [39]. The results of our study showed no significant differences in C-peptide concentrations in the umbilical cord blood between the three study groups. This confirms, in line with the results of insulin levels in umbilical cord blood, the integrity of fetal ß-cells even in hyperglycemic pregnancies of our study.

The functional experiments in this study demonstrated a specific vasomotor response of fetal placental vessels to different agents. A vasoconstriction as well as vasodilatation of these vessels could be shown following a newly established protocol. An altered vascular function in fetal vessels was detected by substance P–induced vasodilation in patients with insulin-treated GDM. Substance P (SuP) is a tachykinin neuropeptide that mediates a variety of biological effects, including the regulation of blood flow, smooth muscle cell activity, and pain transmission. It also has stimulating functions on the immune system of the digestive tract [40]. Substance P binds to the neurokinin 1 receptor (NK1R), also known as tachykinin receptor 1 (TACR1). Animal models have shown that TACR1 expression in the smooth muscle cells of the uterus is lower in early pregnancy and reaches its maximum at the end of pregnancy [41]. SuP receptors were also detected in the trophoblast of fetal villi in human placentas and were suggested to play a role in maintaining placental blood flow [40]. Both preeclampsia [40] and gestational diabetes mellitus resulted in impaired fetoplacental blood flow [42]. The results of this study confirmed an impaired vasorelaxation in fetal placental vessels from women with insulin-treated gestational diabetes compared to normoglycemic controls. This was supported by a reduced substance P receptor (*TACR1*) mRNA expression in fetal placental vessels from patients with iGDM. Substance P is known to induce relaxation in an endothelium-dependent manner [43] suggesting an endothelial dysfunction in the fetal vessels from placentas of women with iGDM. McElwain et al. proposed that such an endothelial dysfunction could have a significant effect on the long-term maternal cardiometabolic health outcomes of both the mother and the child after gestational diabetes mellitus [44].

The best treatment option of gestational diabetes is currently lifestyle changes. A behavioral intervention addressing diet and physical activity in women with obesity during pregnancy (UPBEAT RCT) was not adequate to prevent gestational diabetes or to reduce the incidence of large-for-gestational-age infants [45]. However, several follow-up studies showed numerous beneficial effects of this behavioral (diet and exercise) intervention including improvement of lipids and lipoprotein profile [46], lower offspring pulse rate, and sustained improvement in maternal diet [47] and improved the methylation signatures associated with maternal dysglycemia epigenome of the infants [48]. The UPBEAT intervention also improved cardiac structure and function, preventing concentric remodeling of the infant’s heart at 3 years of age [49]. In conclusion, improved control of diet and physical activity might have also beneficial effects on endothelial function in women with gestational diabetes.

Furthermore, our results support clinical and functional differences between patients with iGDM and dGDM. This supports also different pathophysiological mechanisms underlying these two major subtypes of GDM and might open novel preventive and therapeutic options for the most common pregnancy-associated disease worldwide with its additional long-term cardiometabolic complications for the mother and the child.

## Study limitations

The present study analyzed a limited number of 77 placentas. For optimized experimental protocols, only placentas from caesarean sections were used. Due to the clinical indications for caesarean sections, placentas from this control group could represent higher-risk pregnancies and might not be a completely unbiased control group [50].

## Supplementary Information

Below is the link to the electronic supplementary material.

Supplementary file1 (DOCX 218 kb)

## Data Availability

All datasets as well as relevant information about methods and used materials are documented, saved electronically, and managed by Heike Brendel and Henning Morawietz at Division of Vascular Endothelium and Microcirculation, Department of Medicine III, University Hospital Carl Gustav Carus Dresden, TUD Dresden University of Technology, Dresden, Germany. Datasets will be available upon reasonable request. For any requests, please contact the corresponding author.
