# Correction to: Characterization of human placental fetal vessels in gestational diabetes mellitus

**DOI:** 10.1007/s00424-024-03052-6

**Published:** 2024-12-11

**Authors:** Philine S. Carstens, Heike Brendel, M. Leyre Villar-Ballesteros, Jennifer Mittag, Clara Hengst, Coy Brunssen, Cahit Birdir, Paul D. Taylor, Lucilla Poston, Henning Morawietz

**Affiliations:** 1 Division of Vascular Endothelium and Microcirculation, Department of Medicine III, Faculty of Medicine and University Hospital Carl Gustav Carus, TUD Dresden University of Technology, Fetscherstr. 74, 01307 Dresden, Germany; 2 Department of Obstetrics and Gynecology, Faculty of Medicine and University Hospital Carl Gustav Carus, TUD Dresden University of Technology, Dresden, Germany; 3 Center for Feto/Neonatal Health, Faculty of Medicine and University Hospital Carl Gustav Carus, TUD Dresden University of Technology, Dresden, Germany; 4 Department of Women & Children’s Health, School of Life Course & Population Sciences, King’s College London, London, UK

**Correction to: Pflügers Archiv-European Journal of Physiology**

10.1007/s00424-024-03028-6

In the original version of this manuscript, there are 9 authors listed in the author group, which are Philine S. Carstens, Heike Brendel, M. Leyre Villar Ballesteros, Jennifer Mittag, Clara Hengst, Cahit Birdir, Paul D. Taylor, Lucilla Poston, and Henning Morawietz, and the author requested to add one author in the author group, which is Coy Brunssen.

The corrected list in the author group is presented below.

Philine S. Carstens, Heike Brendel, M. Leyre Villar-Ballesteros, Jennifer Mittag, Clara Hengst, Coy Brunssen, Cahit Birdir, Paul D. Taylor, Lucilla Poston, Henning Morawietz

The original article has been corrected.

