# Supplementary material for: Characterization of human placental fetal vessels in gestational diabetes mellitus
Source: Pflugers Arch. 2024 Oct 9;477(1):67–79. doi: 10.1007/s00424-024-03028-6 (PMC11711144; doi:10.1007/s00424-024-03028-6)
Supplement: Supplementary file 1 — Supplementary file1 (DOCX 218 kb) [file 424_2024_3028_MOESM1_ESM.docx]

**Online Resource**

**Title**

**Characterization of human placental fetal vessels in gestational diabetes mellitus**

**Author information**

Philine S. Carstens^1^, Heike Brendel^1^, M. Leyre Villar-Ballesteros^1,4^, Jennifer Mittag^1^, Clara Hengst^1^, Cahit Birdir^2,3^, Paul D. Taylor^4^, Lucilla Poston^4^ and Henning Morawietz^1^

^1^Division of Vascular Endothelium and Microcirculation, Department of Medicine III, ^2^Department of Obstetrics and Gynecology, ^3^Center for Feto/neonatal Health,

Faculty of Medicine and University Hospital Carl Gustav Carus, TUD Dresden University of Technology, Dresden, Germany;

^4^Department of Women & Children’s Health, School of Life Course & Population Sciences, King’s College London, London, UK.

**Corresponding author**

Henning Morawietz, PhD, FAHA, Division of Vascular Endothelium and Microcirculation, Department of Medicine III, Faculty of Medicine and University Hospital Carl Gustav Carus, TUD Dresden University of Technology, Fetscherstr. 74, 01307 Dresden, Germany

Tel: +49 351 458 6625

Fax: +49 351 458 6354

Email: [Henning.Morawietz@tu-dresden.de](mailto:Henning.Morawietz@tu-dresden.de)

**ORCID ID**

Heike Brendel: 0000-0003-1214-1807

Lucilla Poston: [0000-0003-1100-2821](https://orcid.org/0000-0003-1100-2821)

Henning Morawietz: 0000-0001-9360-9736

**Keywords**

Gestational diabetes, Fetal placental vessels, Endothelial function, Substance P

**Online Resource, Total word count** 451

**Online Resource, Total number of figures** 2

**Online Resource, Results**

**Serum analysis of umbilical cord blood**

Concentrations of C-peptide, that is able to cross the placenta, showed no significant differences between the study groups or venous and arterial blood (**Online Resource, Figure 1A-C**).

**Functional analysis revealed alterations in fetal vessels of insulin-dependent gestational diabetic mothers**

The neurotransmitter norepinephrine is a well-described vasoconstrictor in several other human vessels. However, norepinephrine was not able to induce a vasoconstriction in fetal placental vessels (**Online Resource, Figure 2A**). In contrast, the thromboxane analogue U46619 induced a vasoconstriction in fetal vessels of the placenta (**Online Resource, Figure 2B**). However, it was not possible to reverse the contractile effects on the vessel segments by washing. Therefore, U46619 was not used for further functional analysis.

In addition, vasorelaxation with acetylcholine and bradykinin was tested. Acetylcholine was not able to induce a relaxation in the fetal vessels (**Online Resource, Figure 2C**). Similarly, bradykinin has a potent endothelium-dependent vasodilatory effect in several other human vessels. However, in the fetal vessels of the placenta bradykinin did not cause a relaxation (**Online Resource, Figure 2D**).

**Online Resource, Figures**

**
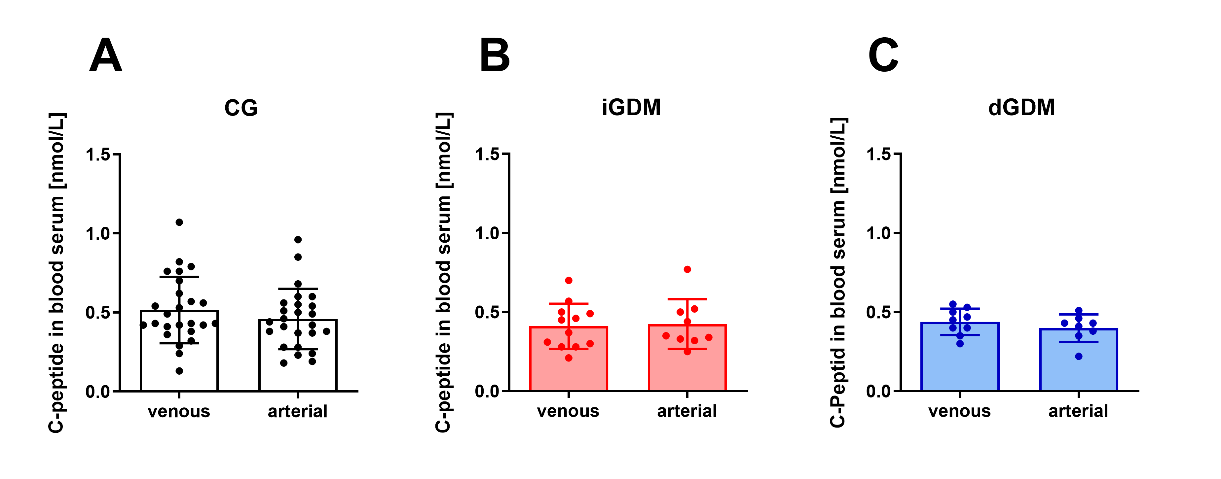
**

**Online Resource, Fig. 1.** **C-peptide concentrations in venous and arterial fetal blood.** Arterial and venous level of C-peptide in **(A)** serum of umbilical cord blood in control group (CG), **(B)** serum of umbilical cord blood in insulin-treated (iGDM) and **(C)** diet-controlled (dGDM) gestational diabetes mellitus patients. Statistics: t-test; p<0.05; n=8–25.

**
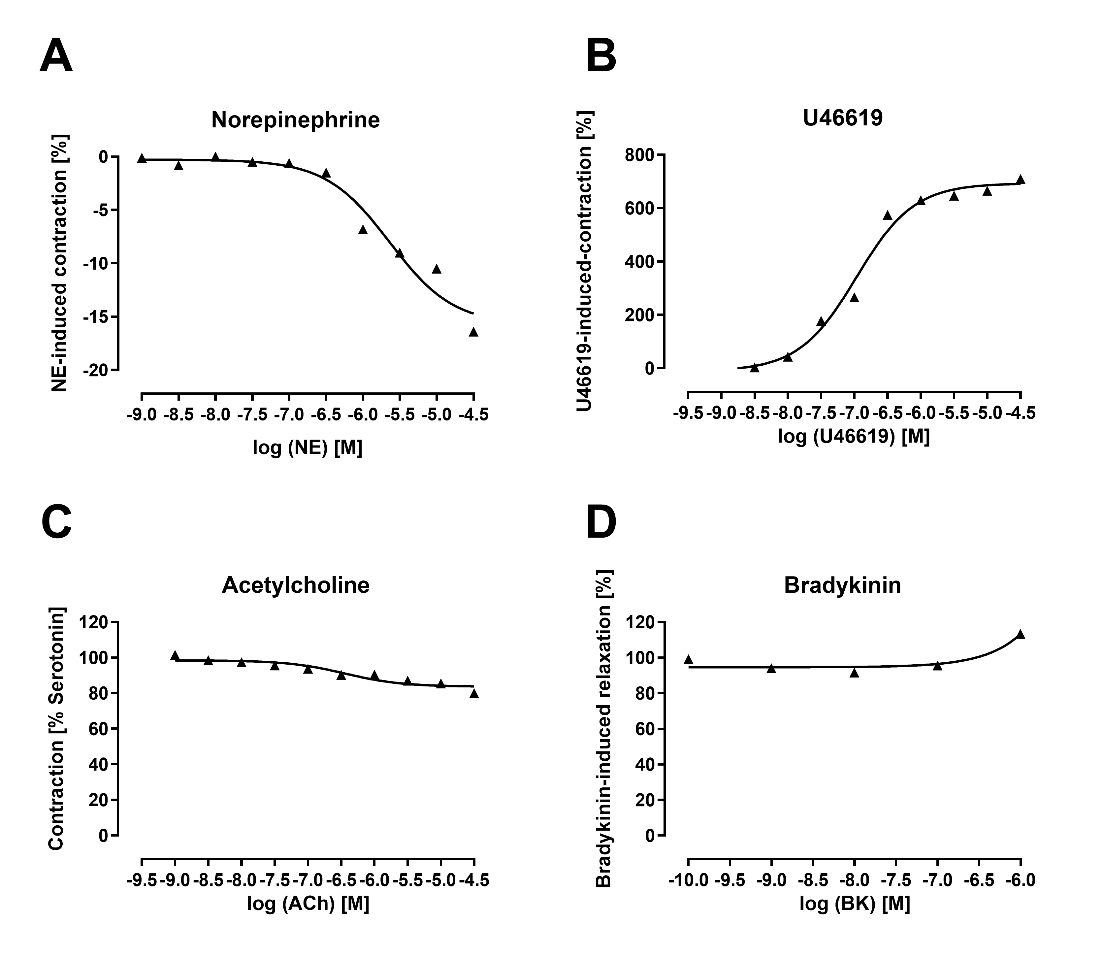
**

**Online Resource, Fig. 2.** **Additional tested substances in fetal placental vessels. (A)** Concentration-response curve of norepinephrine in fetal placental vessels. **(B)** Concentration-response curve of U46619 in fetal placental vessels. **(C)** Concentration-response curve of acetylcholine in serotonin-precontracted fetal placental vessel. **(D)** Concentration-response curve of bradykinin in serotonin-precontracted fetal placental vessels.
